# Supplementary material for: Integrative single-nucleus multi-omics analysis prioritizes candidate cis and trans regulatory networks and their target genes in Alzheimer’s disease brains
Source: Cell Biosci. 2023 Oct 3;13:185. doi: 10.1186/s13578-023-01120-5 (PMC10546724; doi:10.1186/s13578-023-01120-5)
Supplement: Supplementary file 1 — Additional file 1: Figure S1. Confirmation of cluster annotation. a, Feature plots of log-normalized, corrected count data from SCTransform output showing cell type-specific markers for astrocytes (SLC1A2, AQP4, GFAP), neurons (RBFOX3), excitatory neurons (SLC17A7), inhibitory neurons (GAD1, GAD2, SLC6A1), microglia (APBB1IP, C3, CD74, CSF1R), oligodendrocytes (MOBP), and OPCs (MEGF11), and endothelial cell markers (FLT1, CLDN5) as negative controls. b, Chromatin accessibility tracks for gene promoter and coding regions for cell type markers for oligodendrocytes (MOBP), microglia (C3), astrocytes (AQP4), excitatory neurons (SLC17A7), OPCs (MEGF11), and inhibitory neurons (GAD2). c, UMAP plots showing cell type annotation based on snRNA-seq of human primary motor cortex [33] (left) and snRNA-seq of human prefrontal cortex [36] (right), d, UMAP plot of re-annotated snRNA-seq dataset with known excitatory and inhibitory neuron subtypes. e, Proportions of nuclei of each of the five subtypes of excitatory and inhibitory neurons for each sample. Average proportions for the 12 LOAD and 12 Normal samples are shown. Figure S2. Distribution of nuclei among cell subtype clusters by donor sample ID. a, UMAP dimensional reduction plots of cell subtype clusters for snRNA-seq dataset split by donor sample ID. b, UMAP plots of cell subtype clusters for snATAC-seq dataset split by donor sample ID. Cell subtype clusters are color coded. Figure S3. Correlation of metadata covariates in snRNA-seq and snATAC-seq data. Figure S4. Top differentially-expressed genes (DEGs) upregulated and down-regulated in LOAD by cluster. Unbiased volcano plots for all clusters containing DEGs not shown in Fig. 3, representing astrocyte (Astro), excitatory neuron (Exc), inhibitory neuron (Inh), microglia (Micro), oligodendrocyte (Oligo), and oligodendrocyte precursor (OPC) cell types. Log2 fold change (FC) between LOAD and normal control samples is plotted against –log10 p-value (FDR). Points representing [file 13578_2023_1120_MOESM1_ESM.pdf]

**Figure S1. Confirmation of cluster annotation.**

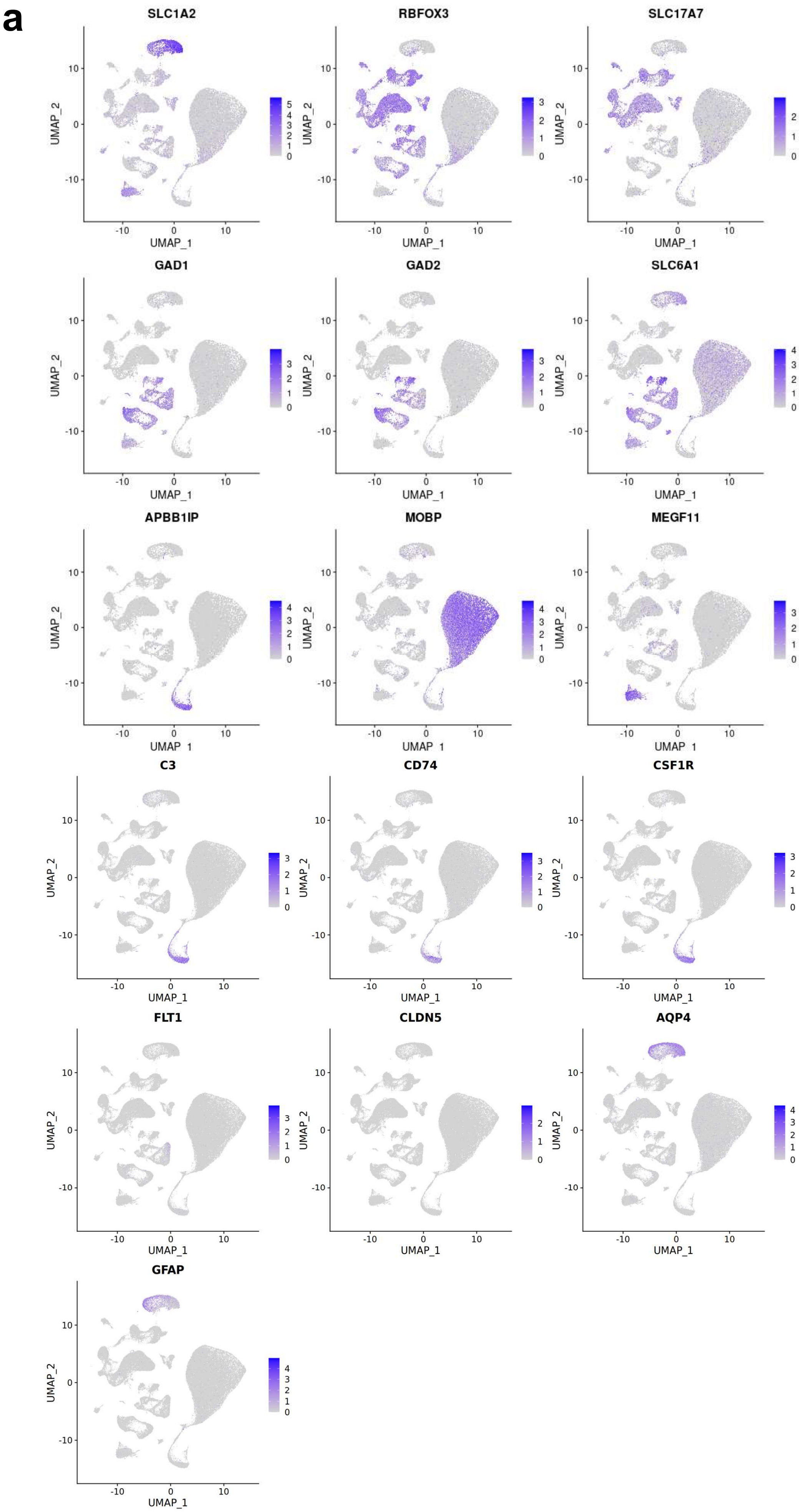

**Figure S1. Confirmation of cluster annotation.**

**b**

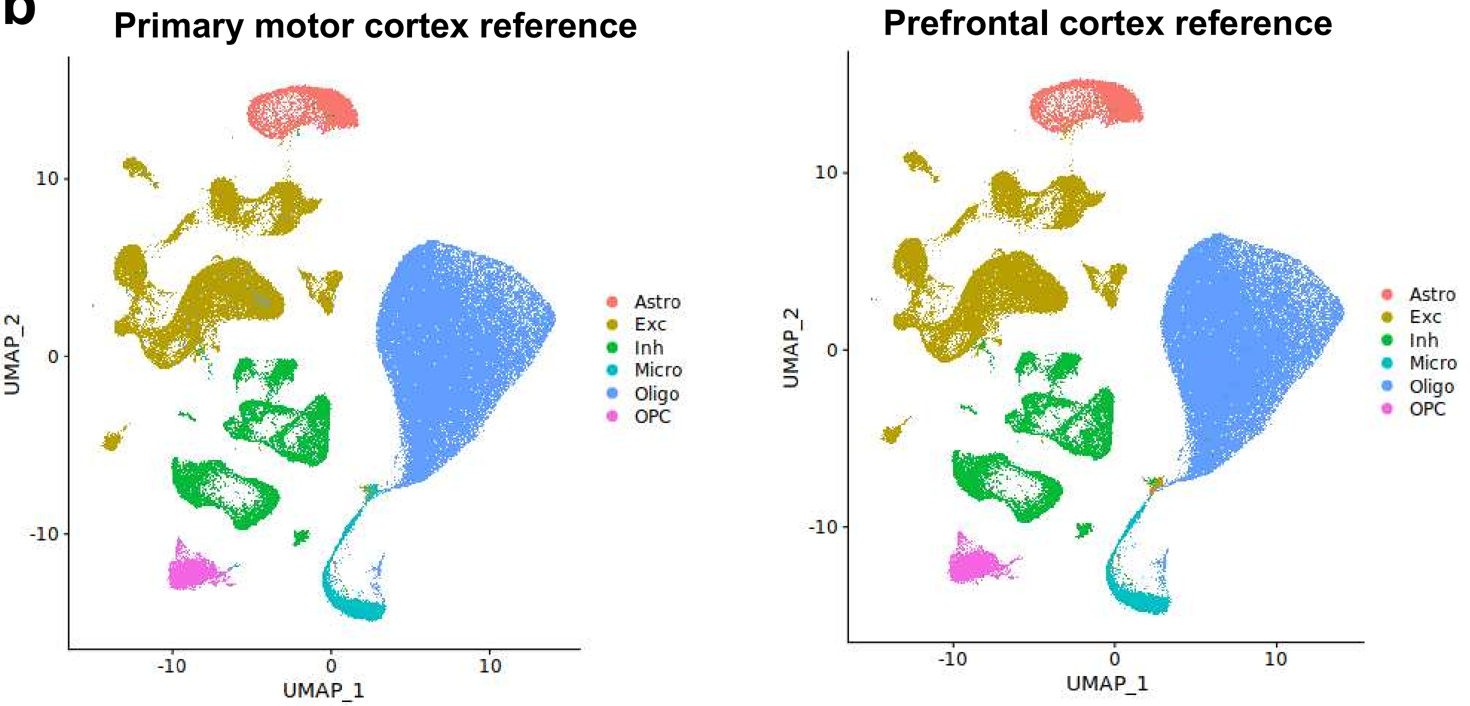


**c**

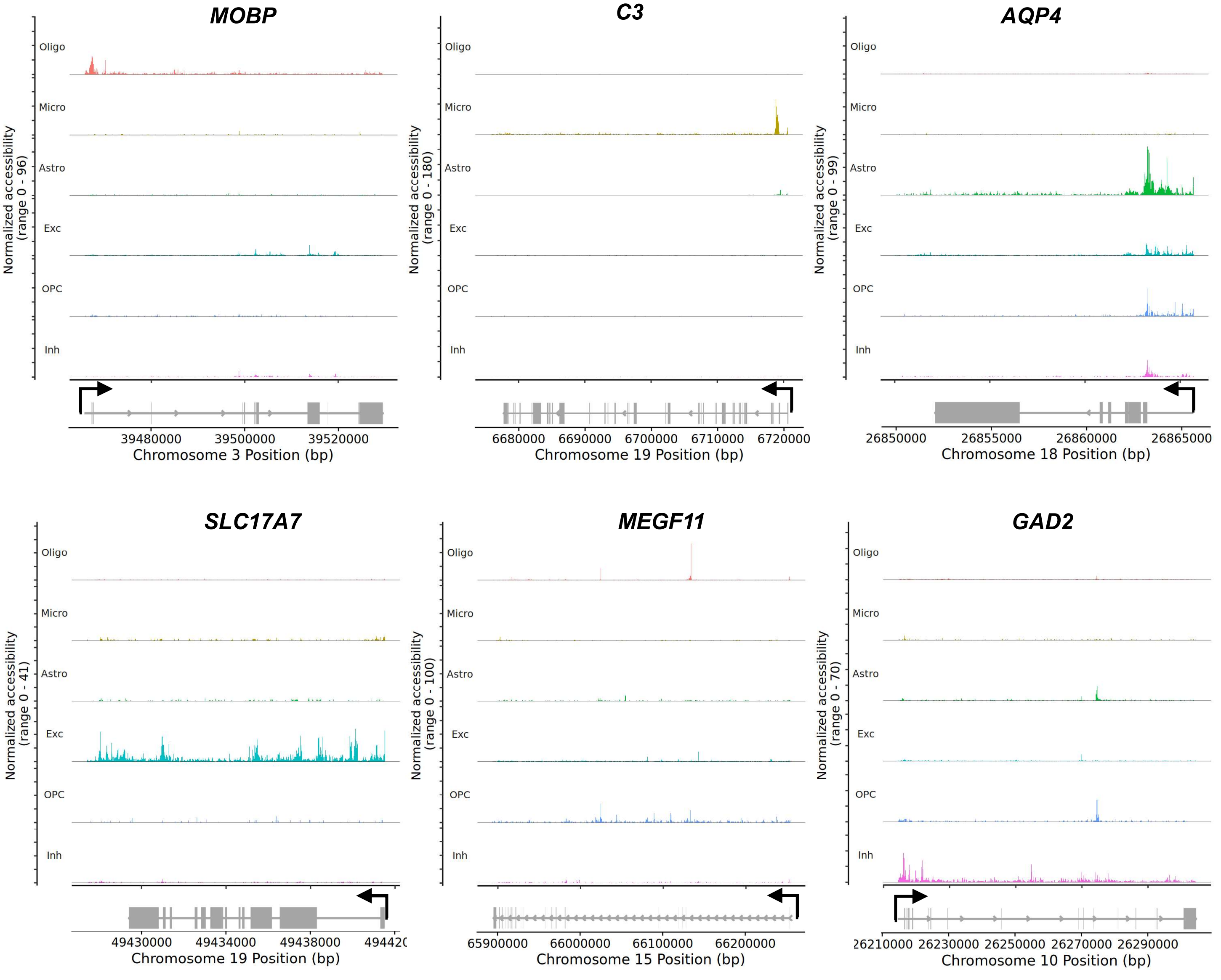

**Figure S1. Confirmation of cluster annotation.**

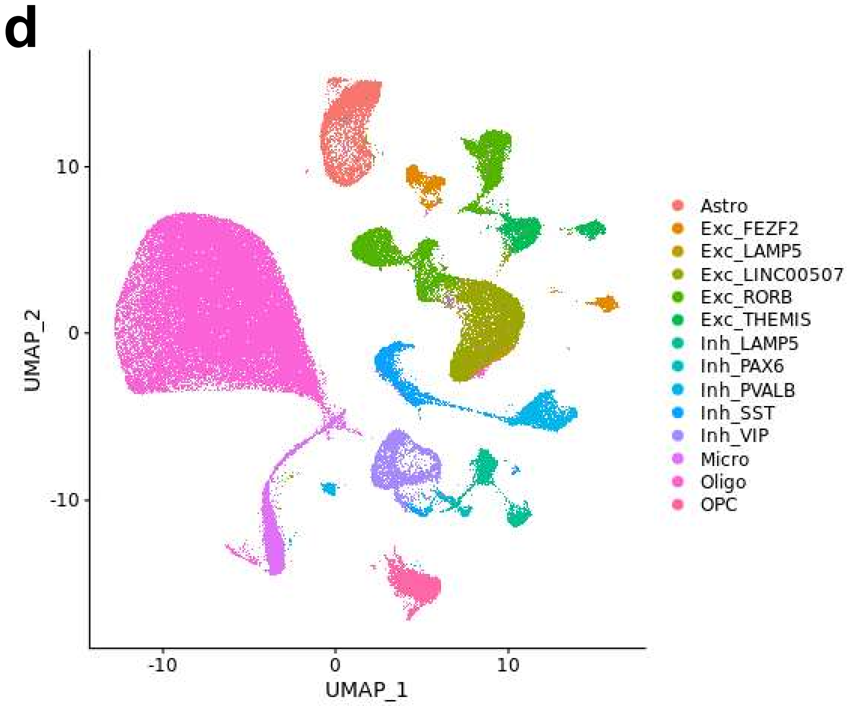


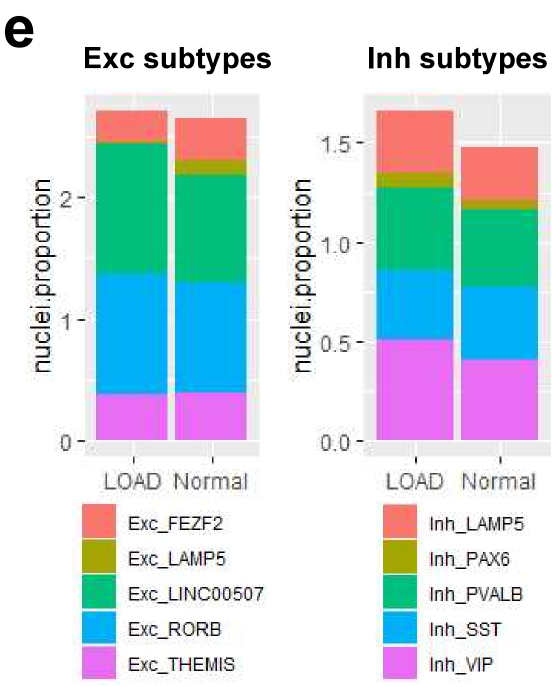

**Figure S1. Confirmation of cluster annotation. a,** Feature plots of log-normalized, corrected count data from SCTransform output showing cell type-specific markers for astrocytes (*SLC1A2, AQP4, GFAP*), neurons (*RBFOX3*), excitatory neurons (*SLC17A7*), inhibitory neurons (*GAD1, GAD2, SLC6A1*), microglia (*APBB1IP, C3, CD74, CSF1R*), oligodendrocytes (*MOBP*), and OPCs (*MEGF11*), and endothelial cell markers (*FLT1*, *CLDN5*) as negative controls. **b**, Chromatin accessibility tracks for gene promoter and coding regions for cell type markers for oligodendrocytes (*MOBP*), microglia (*C3*), astrocytes (*AQP4*), excitatory neurons (*SLC17A7*), OPCs (*MEGF11*), and inhibitory neurons (*GAD2*). **c**, UMAP plots showing cell type annotation based on snRNA-seq of human primary motor cortex[33] (left) and snRNA-seq of human prefrontal cortex[36] (right), **d**, UMAP plot of re-annotated snRNA-seq dataset with known excitatory and inhibitory neuron subtypes. **e**, Proportions of nuclei of each of the five subtypes of excitatory and inhibitory neurons for each sample. Average proportions for the 12 LOAD and 12 Normal samples are shown.

# Figure S2 . Distribution of nuclei among cell subtype clusters by donor sample ID

**a**

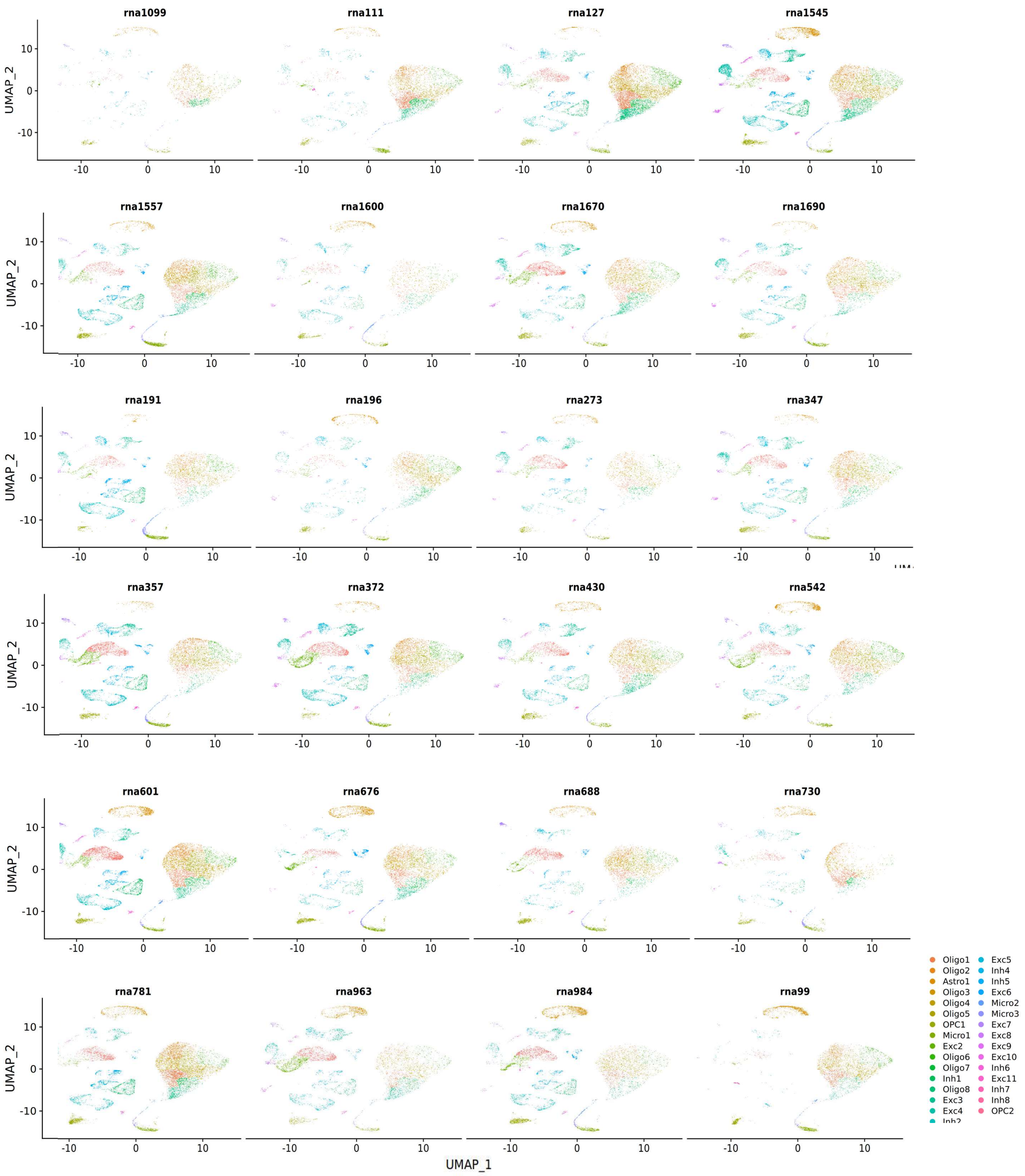

# Figure S2 . Distribution of nuclei among cell subtype clusters by donor sample ID

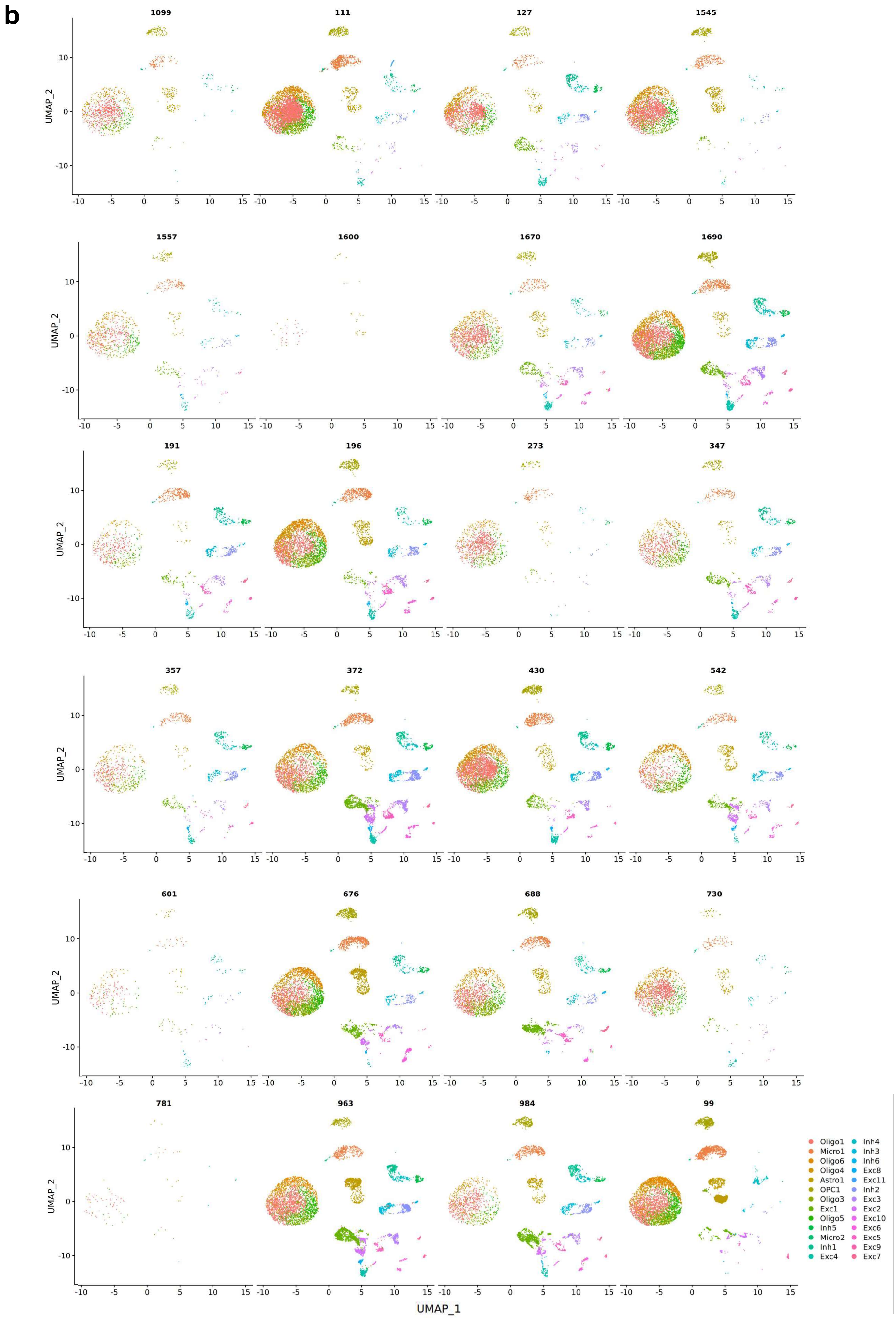

**Figure S2 . Distribution of nuclei among cell subtype clusters by donor sample ID. a,** UMAP dimensional reduction plots of cell subtype clusters for snRNA-seq dataset split by donor sample ID. **b,** UMAP plots of cell subtype clusters for snATAC-seq dataset split by donor sample ID. Cell subtype clusters are color coded.

**Figure S3. Correlation of metadata covariates in snRNA-seq and snATAC-seq data.**

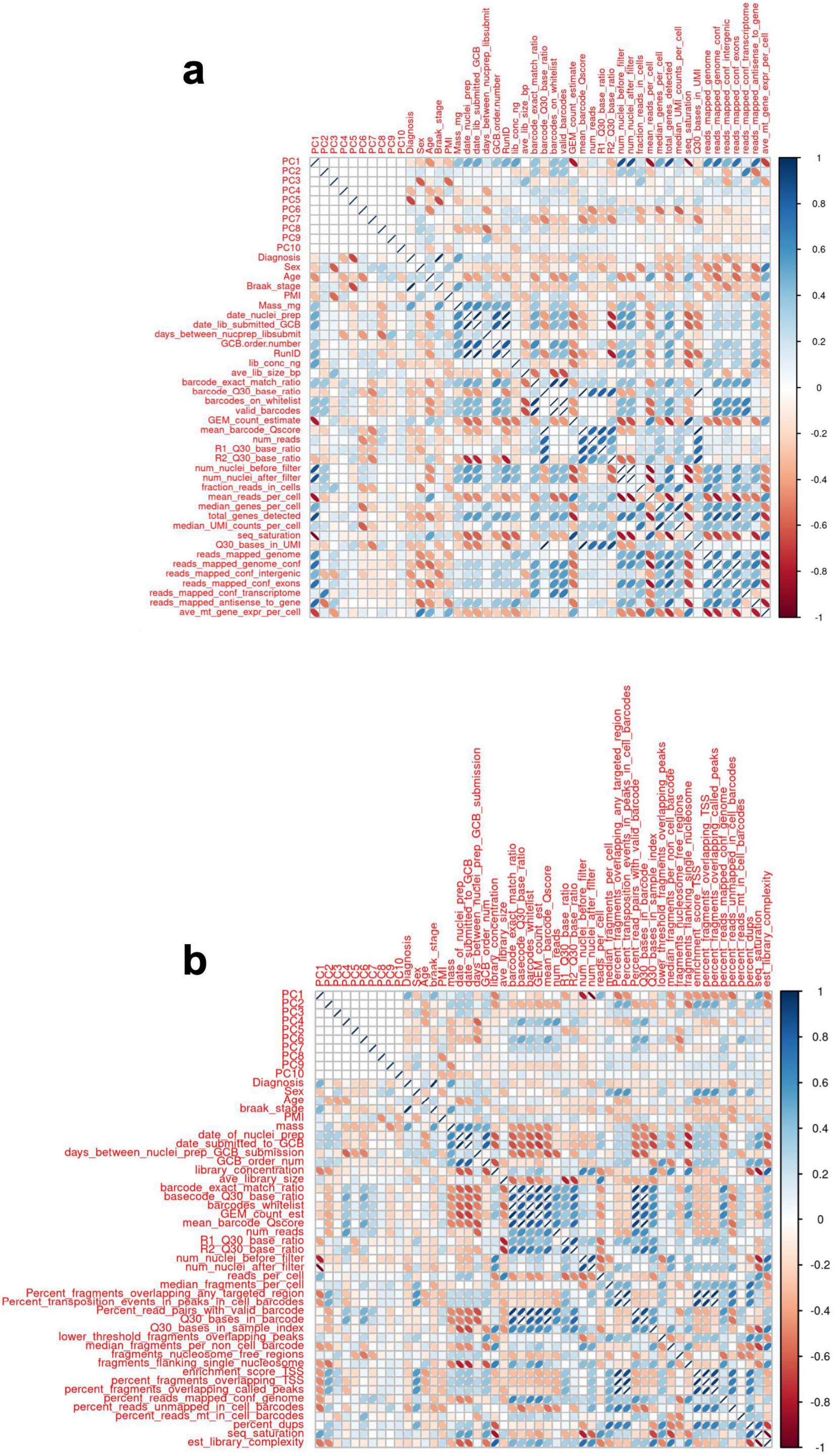

## Figure S4. Top differentially-expressed genes (DEGs) upregulated and down-regulated in LOAD by cluster.

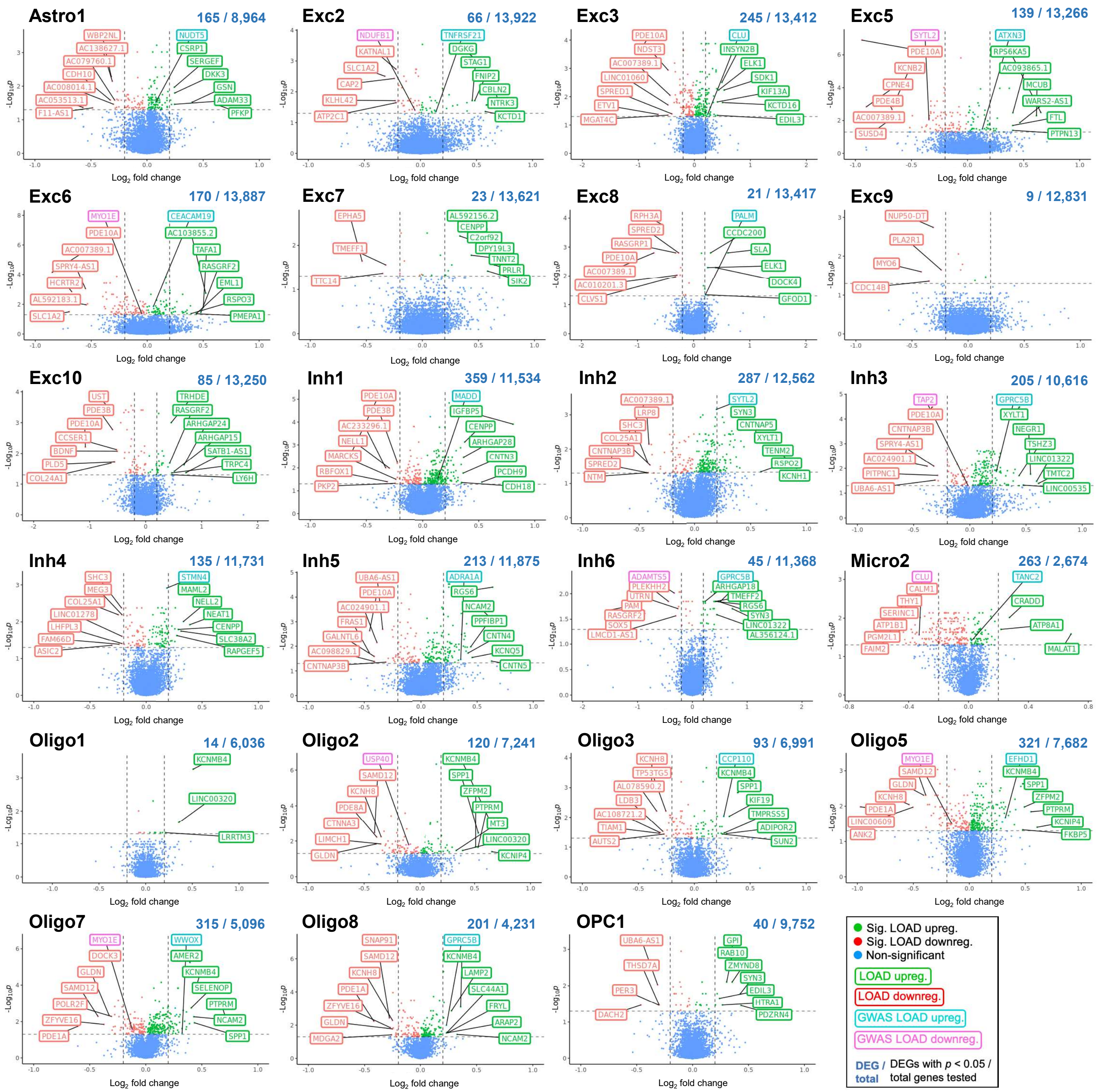

**Figure S4. Top differentially-expressed genes (DEGs) upregulated and down-regulated in LOAD by cluster.** Unbiased volcano plots for all clusters containing DEGs not shown in figure 3, representing astrocyte (Astro), excitatory neuron (Exc), inhibitory neuron (Inh), microglia (Micro), oligodendrocyte (Oligo), and oligodendrocyte precursor (OPC) cell types. $\text{Log}_2$ fold change (FC) between LOAD and normal control samples is plotted against $-\log_{10}$ *p*-value (FDR). Points representing DEGs with statistically significant ($p < 0.05$) upregulation in LOAD are shown in green while DEGs with significant downregulation are shown in red. Genes without significantly differential expression are shown in blue. The proportion of DEGs to total genes examined is shown above each plot. The six DEGs with the highest absolute fold change ($\log_2$ FC > 0.2) in the up- and downregulated categories are labeled in green and red, respectively. The top up- and downregulated DEGs within 500kb of disease-associated SNPs previously identified in GWAS are labeled in teal and pink, respectively. The Inh8 and Inh9 clusters did not contain DEGs and are not shown.

# Figure S5. Gene ontology analysis of DEGs identified in snRNA-seq data for cell types

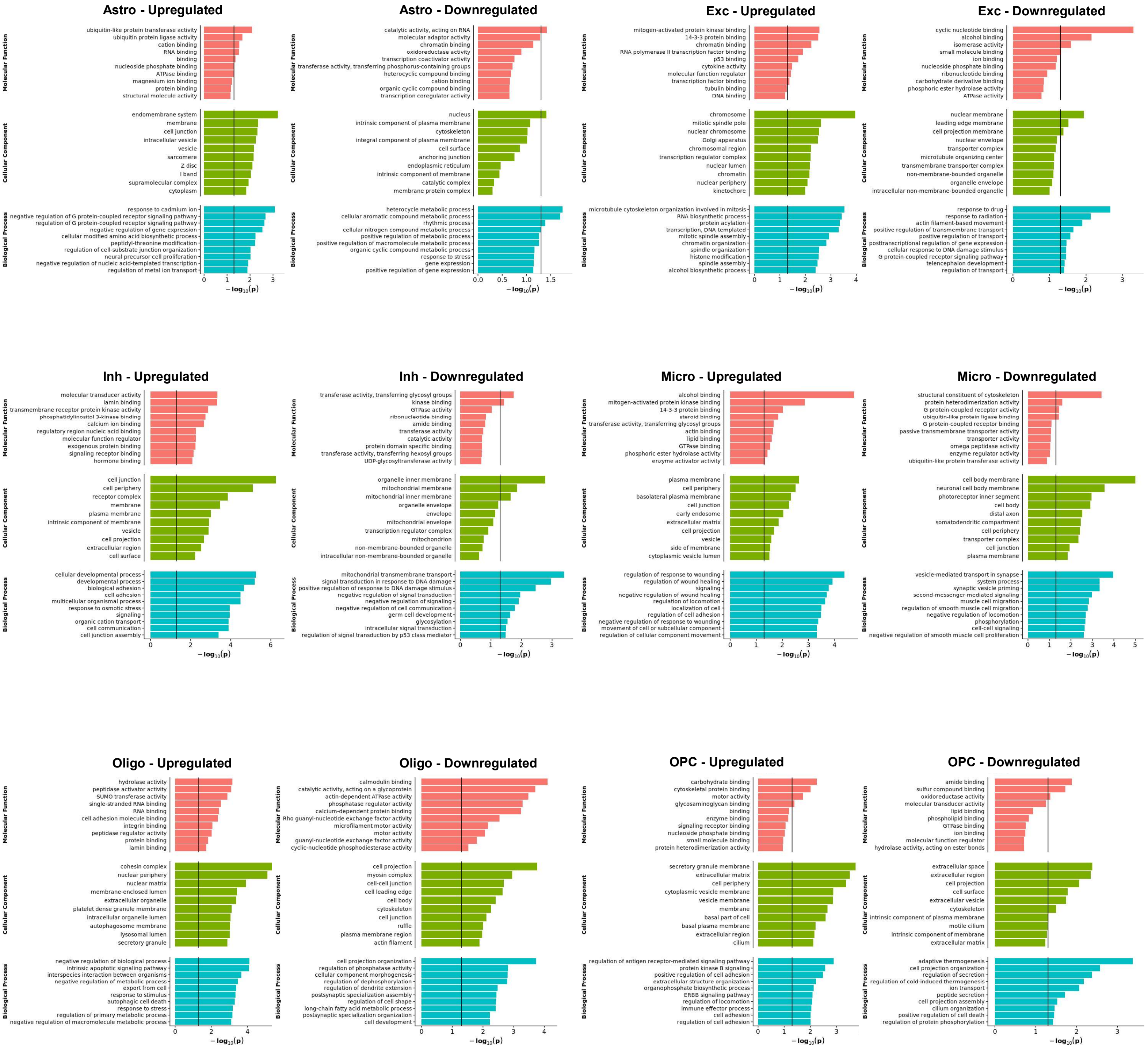

**Figure S5. Gene ontology analysis of DEGs identified in snRNA-seq data for cell types.** Gene ontological analysis of biological processes, cellular components, and molecular functions for DEGs associated with the indicated cell types. Up to the top ten enriched terms involving a minimum of three DEGs are listed. Statistical significance threshold ($p < 0.05$) is indicated by vertical lines.

**Figure S6. Additional gene ontology analysis of cCRE-linked DEGs**

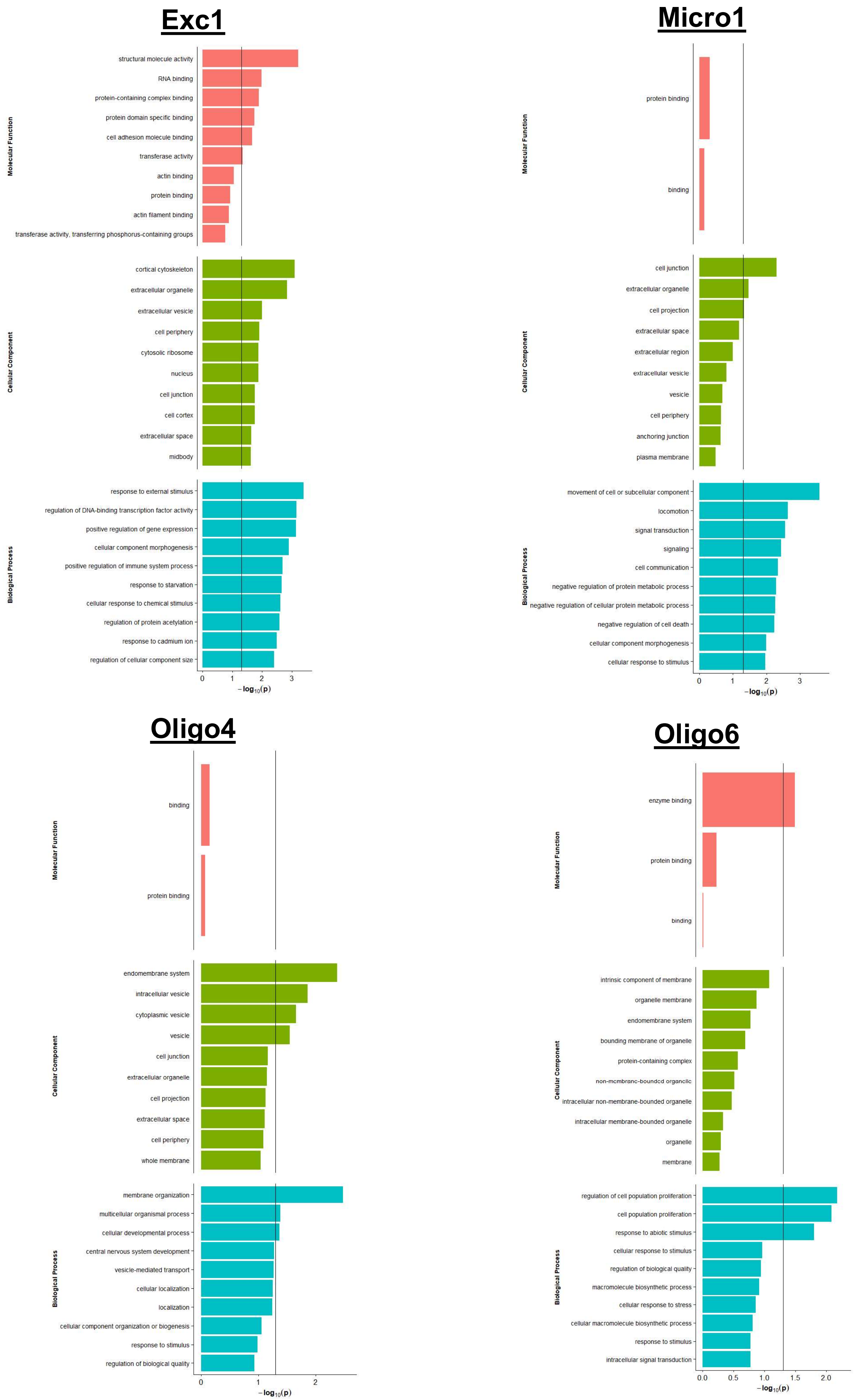

**Figure S6. Additional gene ontology analysis of cCRE-linked DEGs.** Gene ontological analysis of biological processes, molecular functions and cellular components for cCRE-linked DEGs associated with CCANs in the indicated cell subtype clusters. Up to the top ten enriched terms involving a minimum of three DEGs are listed. Statistical significance threshold ($p < 0.05$) is indicated by vertical lines.

**Figure S7. Extended atSNP analysis**

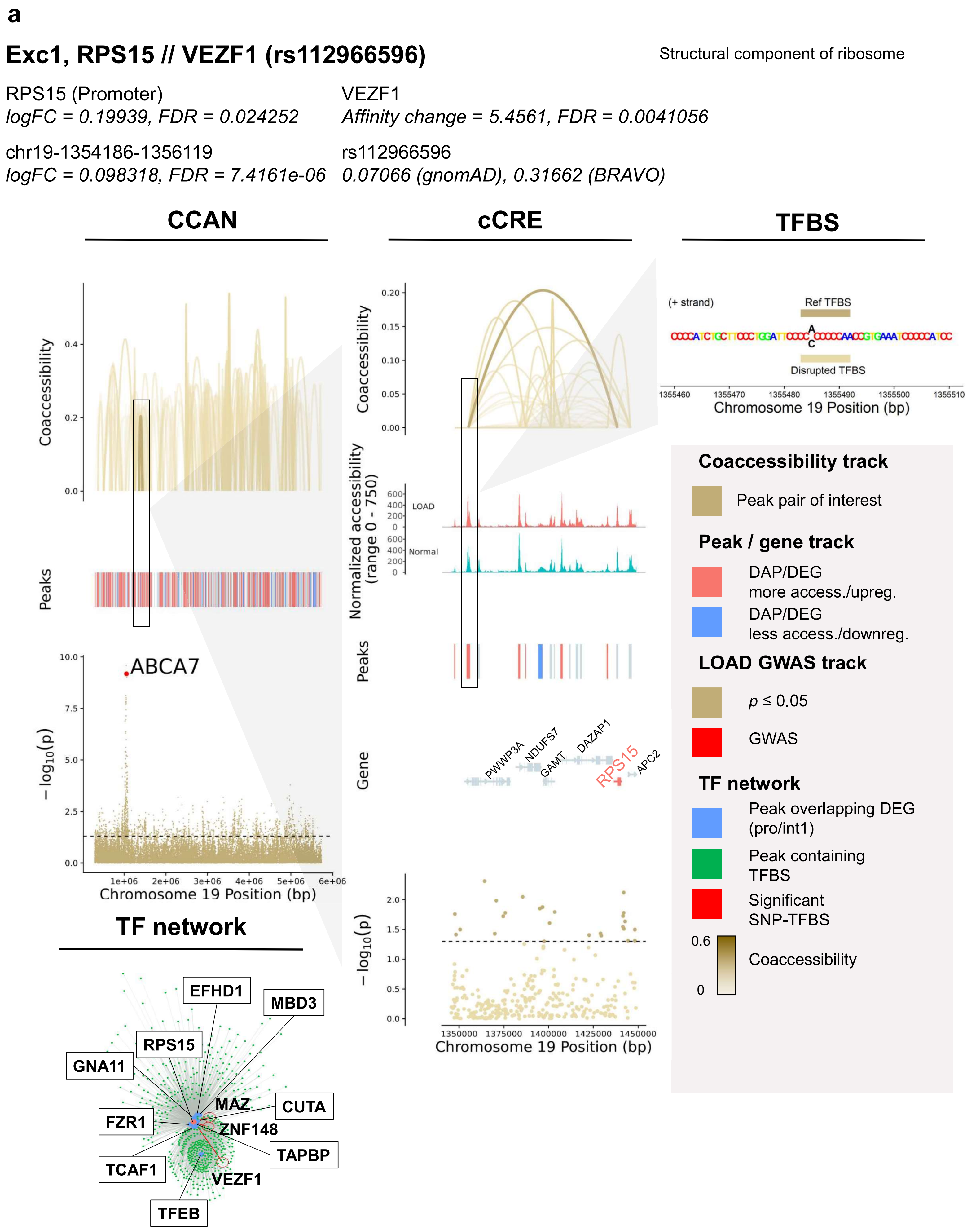

**Figure S7. Extended atSNP analysis**

**b**

## Exc1, RPS15 // ZNF148 (rs114520071)

Structural component of ribosome

RPS15 (Promoter)
*logFC = 0.19939, FDR = 0.024252*

ZNF148
*Affinity change = -4.5576, FDR = 0.0013334*

chr19-1354186-1356119
*logFC = 0.098318, FDR = 7.4161e-06*

rs114520071
*0.000118 (gnomAD), 0.017258 (BRAVO)*

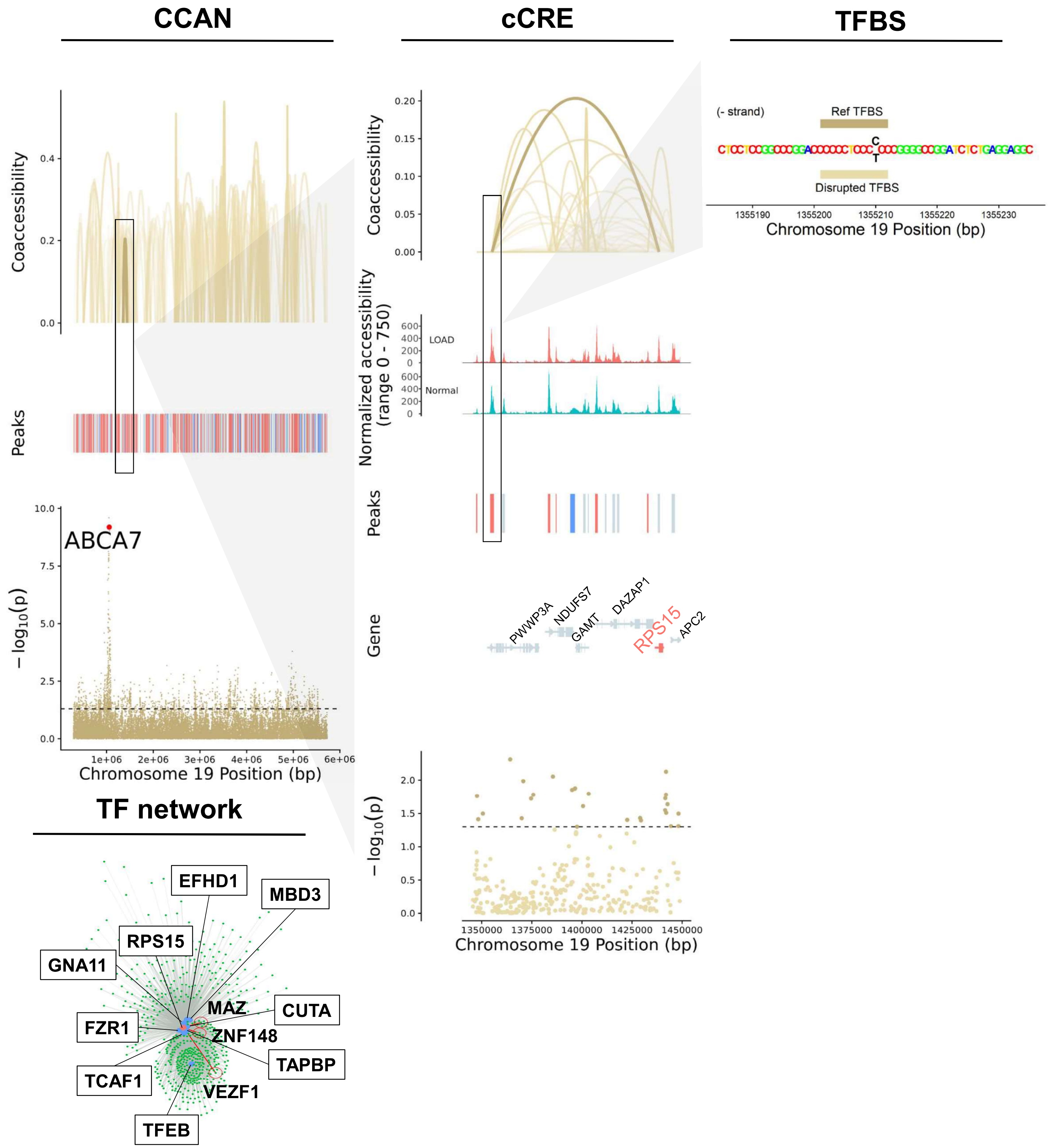

**Figure S7. Extended atSNP analysis**

**c**

## Micro1, MYO1E // TAL1::TCF3 (rs116657283)

Actin-based molecular motors. Involved in phosphatidylinositol binding and calmodulin binding

MYO1E (Promoter + Intron 1)
*logFC = 0.40737, FDR = 0.0012459*

TAL1::TCF3
*Affinity change = -4.5747, FDR = 0.0058775*

chr15-59169011-59170652
*logFC = 0.091773, FDR = 0.0060433*

rs116657283
*0.00008819 (gnomAD), 0.015176 (BRAVO)*

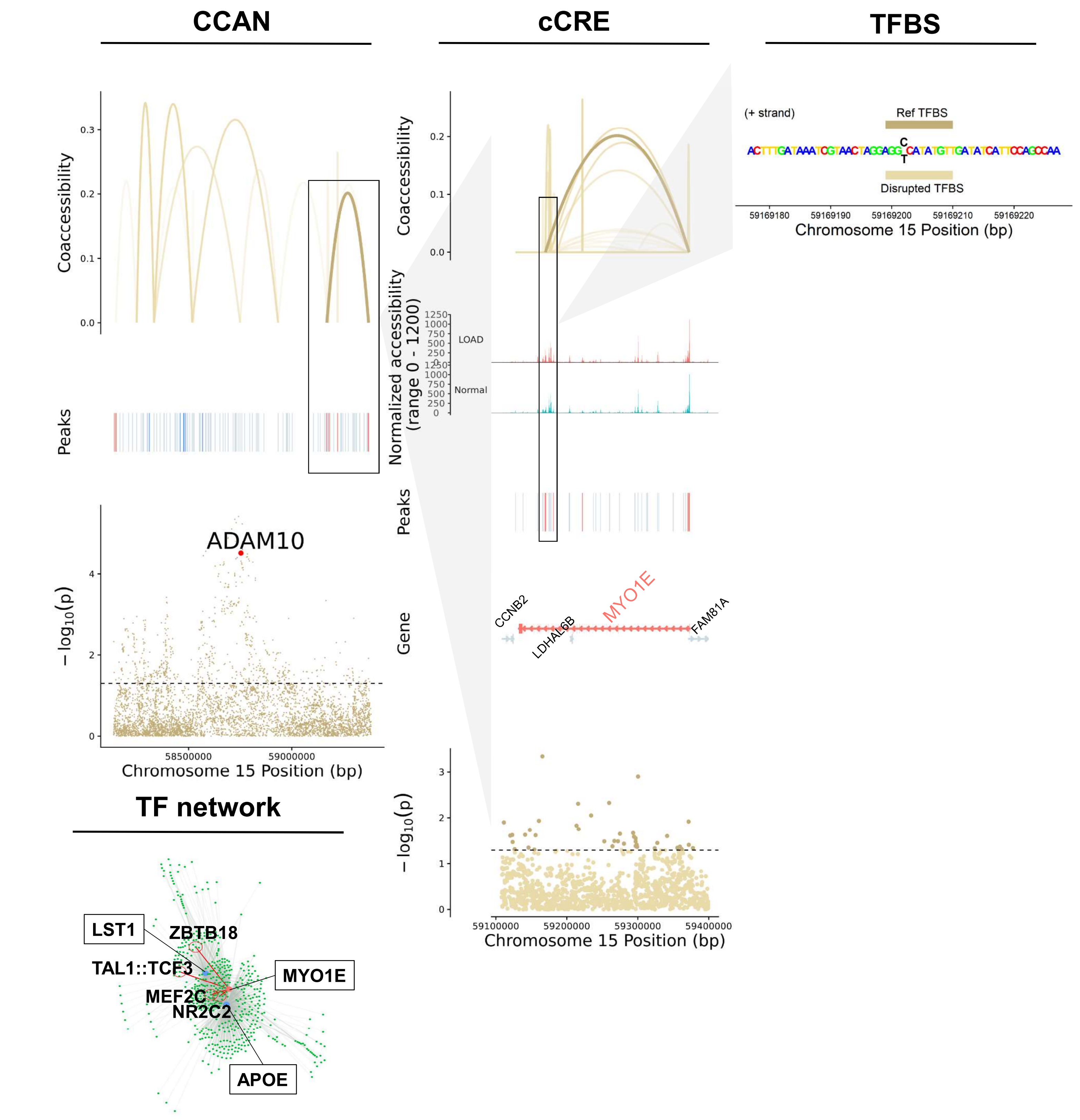

**Figure S7. Extended atSNP analysis**

**d**

## Micro1, MYO1E // TAL1::TCF3 (rs62004105)

Actin-based molecular motors. Involved in phosphatidylinositol binding and calmodulin binding

MYO1E (Promoter + Intron 1)
*logFC = 0.40737, FDR = 0.0012459*

TAL1::TCF3
*Affinity change = -5.1761, FDR = 0.0033228*

chr15-59169011-59170652
*logFC = 0.091773, FDR = 0.0060433*

rs62004105
*0.2338 (gnomAD), 0.34129 (BRAVO)*

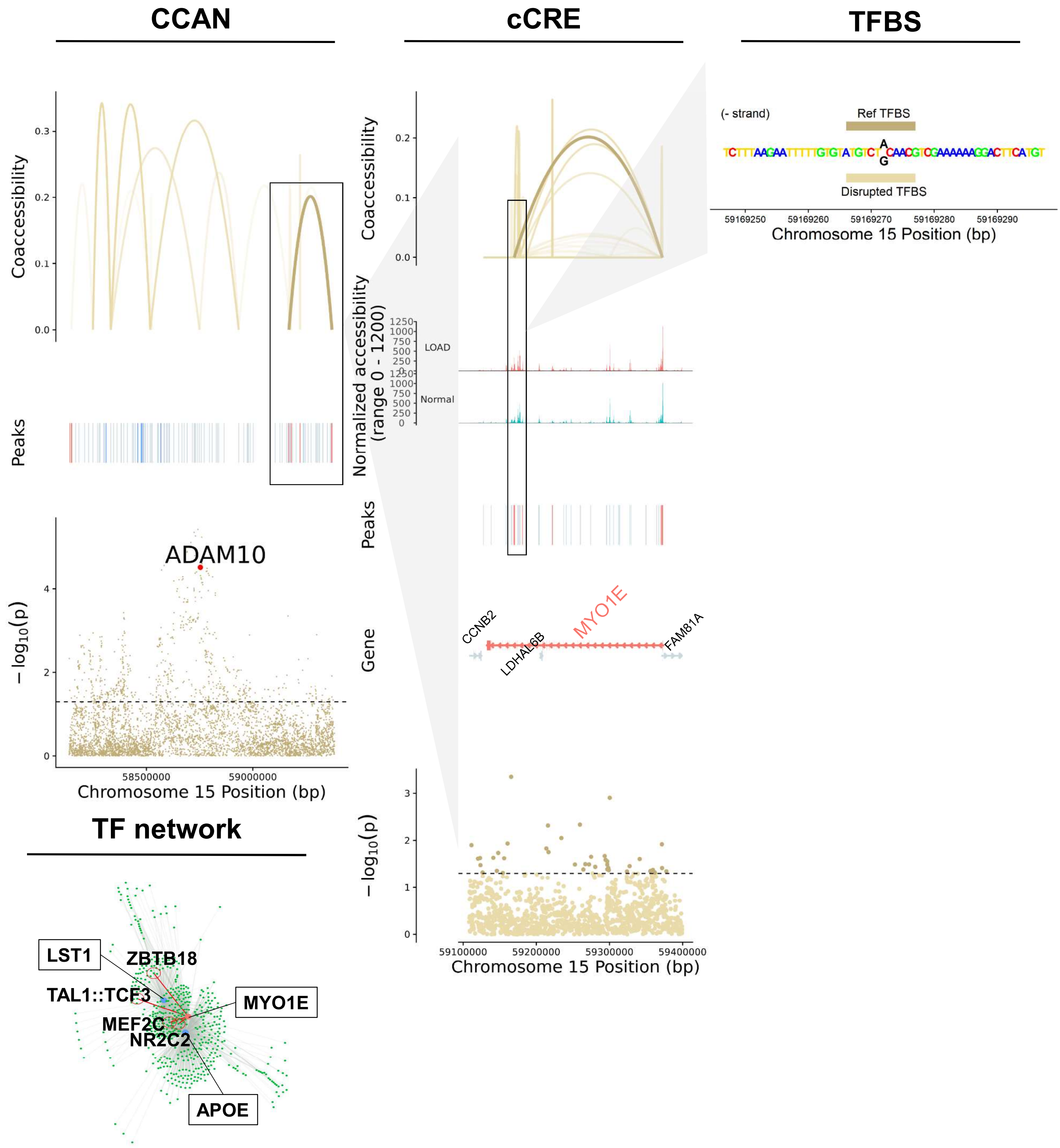

**Figure S7. Extended atSNP analysis**

**e**

## Micro1, MYO1E // ZBTB18 (rs62004105)

Actin-based molecular motors. Involved in phosphatidylinositol binding and calmodulin binding

MYO1E (Promoter + Intron 1)
*logFC = 0.40737, FDR = 0.0012459*

ZBTB18
*Affinity change = -6.7984, FDR = 0.0058473*

chr15-59169011-59170652
*logFC = 0.091773, FDR = 0.0060433*

rs62004105
*0.2338 (gnomAD), 0.34129 (BRAVO)*

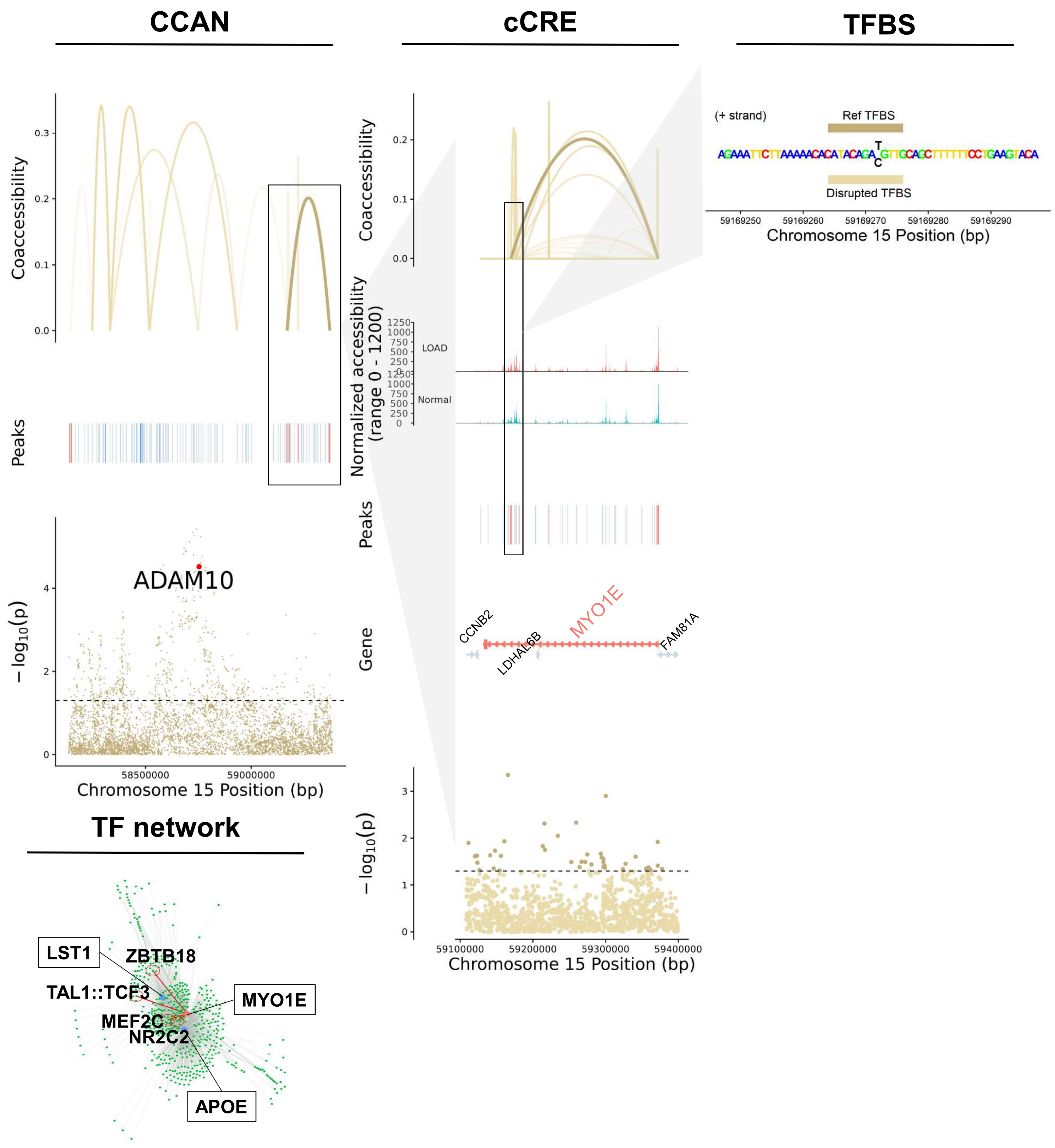

**Figure S7. Extended atSNP analysis**

**f**

## Micro1, MYO1E // MEF2C (rs12438396)

Actin-based molecular motors. Involved in phosphatidylinositol binding and calmodulin binding

MYO1E (Promoter + Intron 1)
*logFC = 0.40737, FDR = 0.0012459*

MEF2C
*Affinity change = -7.8624, FDR = 0.0089648*

chr15-59169011-59170652
*logFC = 0.091773, FDR = 0.0060433*

rs12438396
*0.2276 (gnomAD), 0.29191 (BRAVO)*

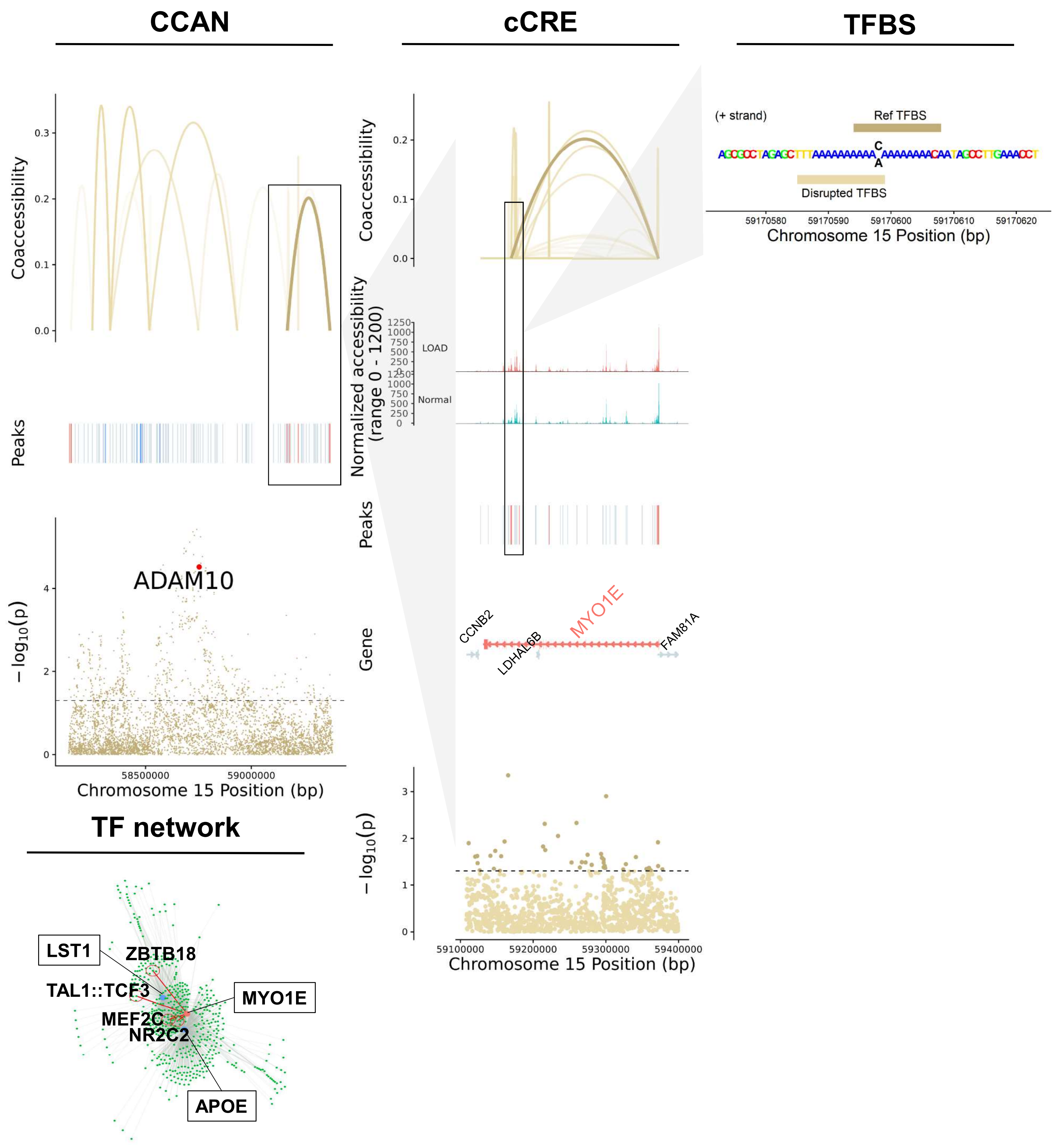

**Figure S7. Extended atSNP analysis**

**g**

## Micro1, APOE // RFX3 (rs35577563)

Functions in lipoprotein lipid transport

APOE (Promoter + intron 1)
*logFC = 0.45447, FDR = 0.0086049*

RFX3
*Affinity change = 4.5593, FDR = 0.0060074*

chr19-45000676-45002343
*logFC = 0.061612, FDR = 7.1923e-05*

rs35577563
*0.3695 (gnomAD), 0.50291 (BRAVO)*

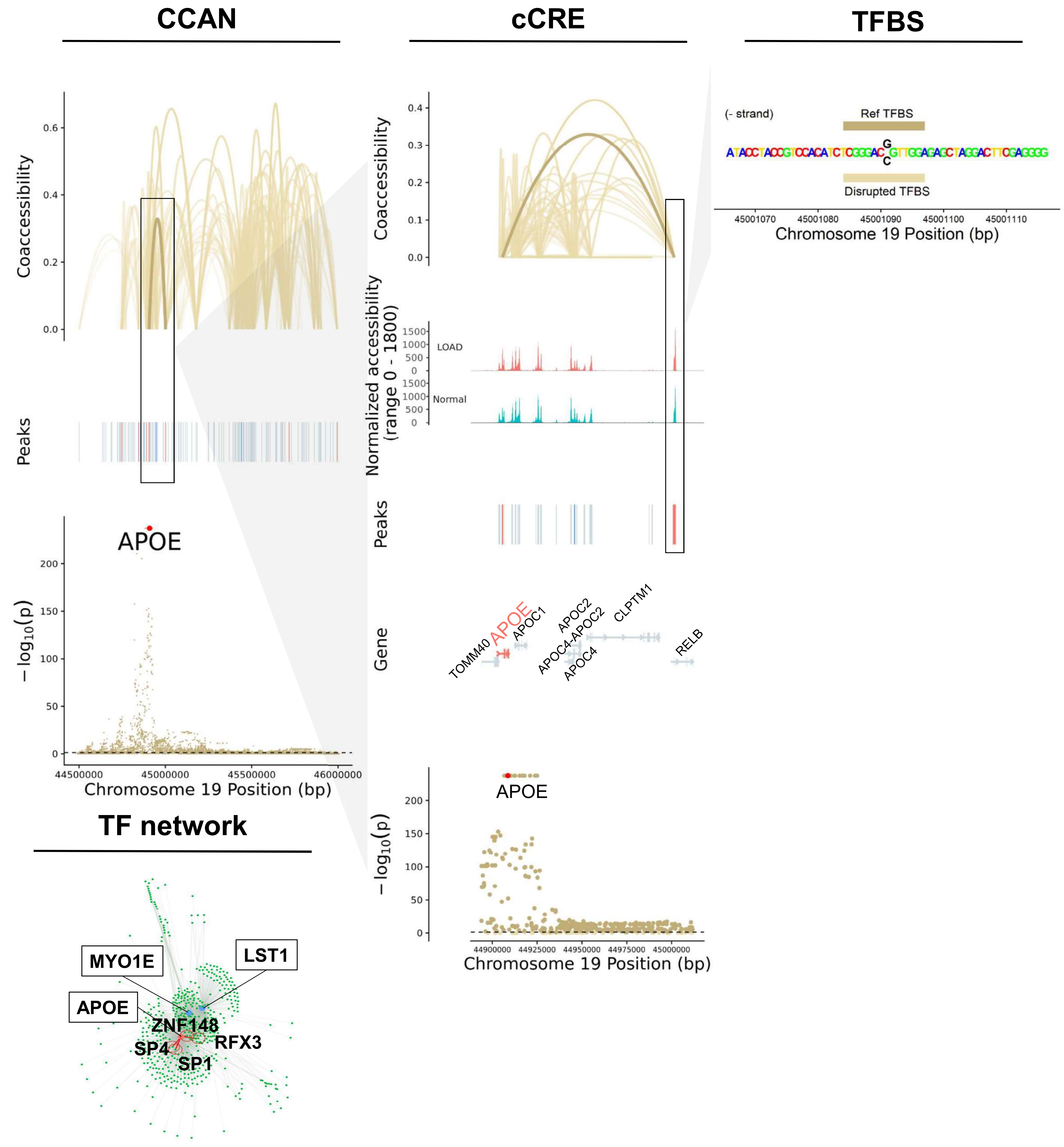

**Figure S7. Extended atSNP analysis**

**h**

## Micro1, APOE // SP4 (rs866159508)

Functions in lipoprotein lipid transport

APOE (Promoter + Intron 1)
*logFC = 0.45447, FDR = 0.0086049*

SP4
*Affinity change = -5.9456, FDR = 0.0026361*

chr19-44845549-44846452
*logFC = 0.051984, FDR = 0.027969*

rs866159508
*0.000207 (gnomAD), 0.021474 (BRAVO)*

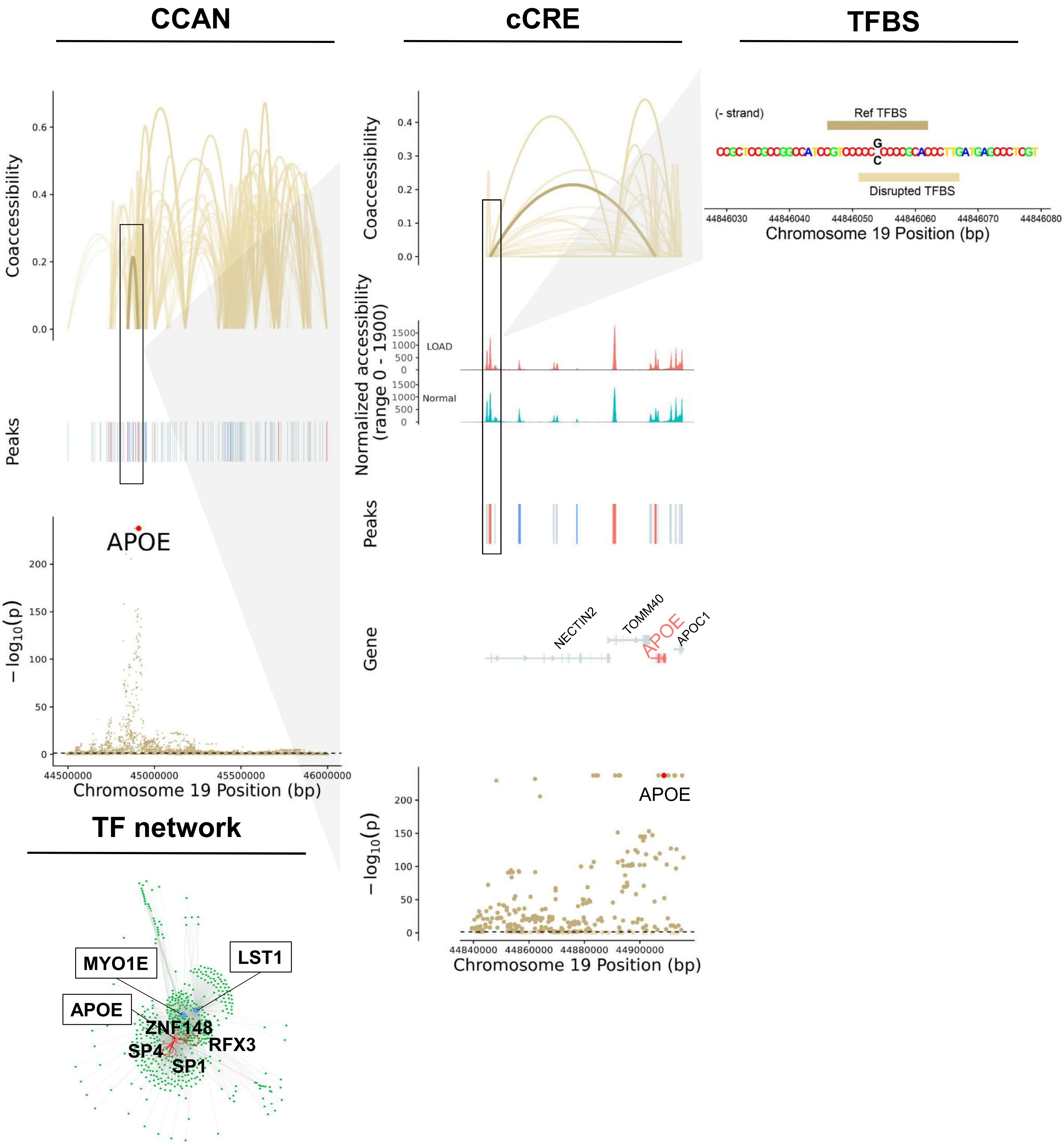

**Figure S7. Extended atSNP analysis**

**i**

## Micro1, APOE // ZNF148 (rs866159508)

Functions in lipoprotein lipid transport

APOE (Promoter + Intron 1)
*logFC = 0.45447, FDR = 0.0086049*

ZNF148
*Affinity change = -4.2632, FDR = 0.0020921*

chr19-44845549-44846452
*logFC = 0.051984, FDR = 0.027969*

rs866159508
*0.000207 (gnomAD), 0.021474 (BRAVO)*

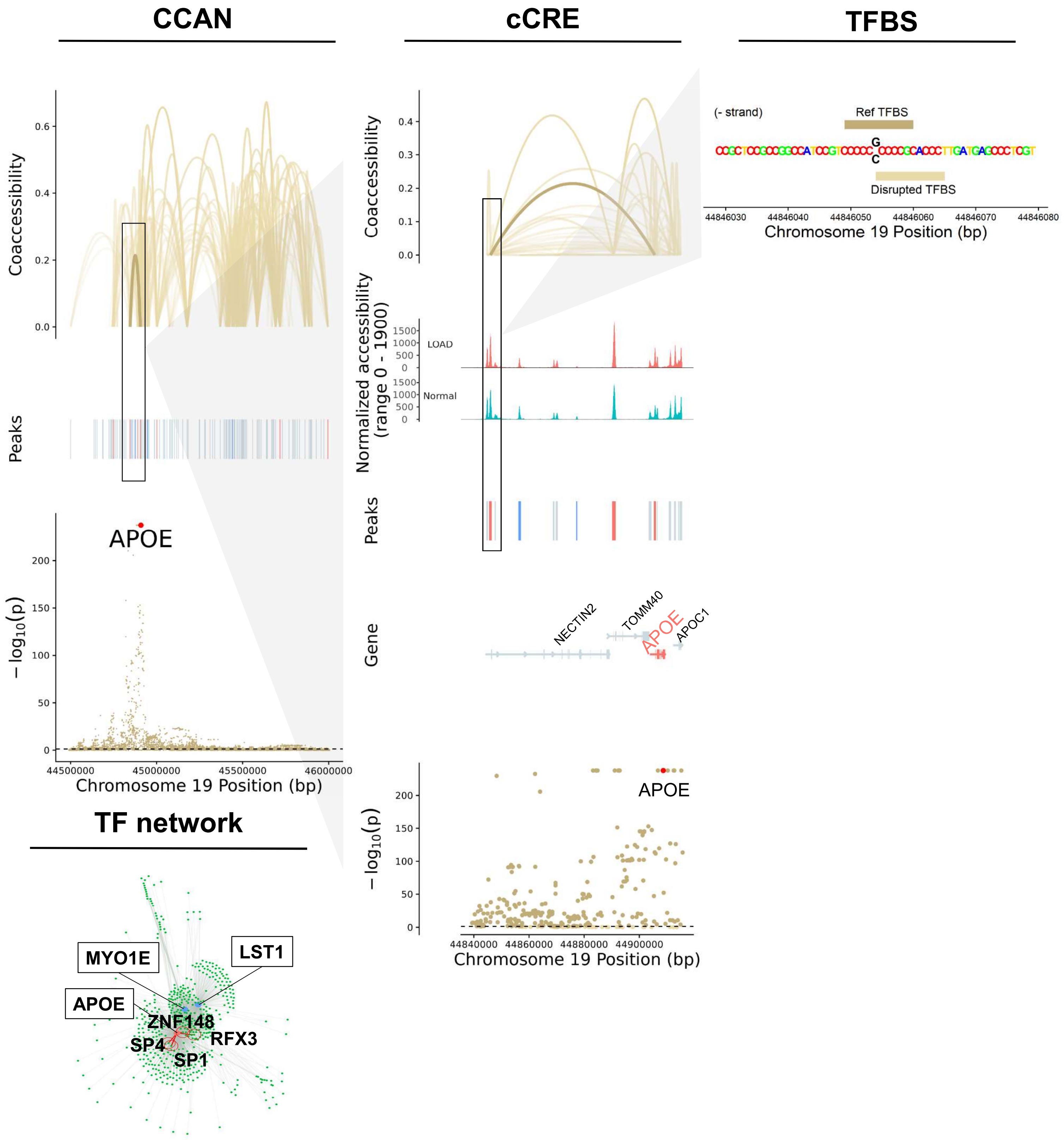

**Figure S7. Extended atSNP analysis**

**j**

## Oligo4, BIN1 // GABPA (rs17014873)

Regulates membrane activity. Involved in cell cycle regulation and aptoptosis

BIN1 (Intron 1)
*logFC = 0.15359, FDR = 0.036918*

GABPA
*Affinity change = 4.0927, FDR = 0.0088645*

chr2-127072401-127073470
*logFC = 0.094153, FDR = 2.5135e-08*

rs17014873
*0.06606 (gnomAD), 0.1158 (BRAVO)*

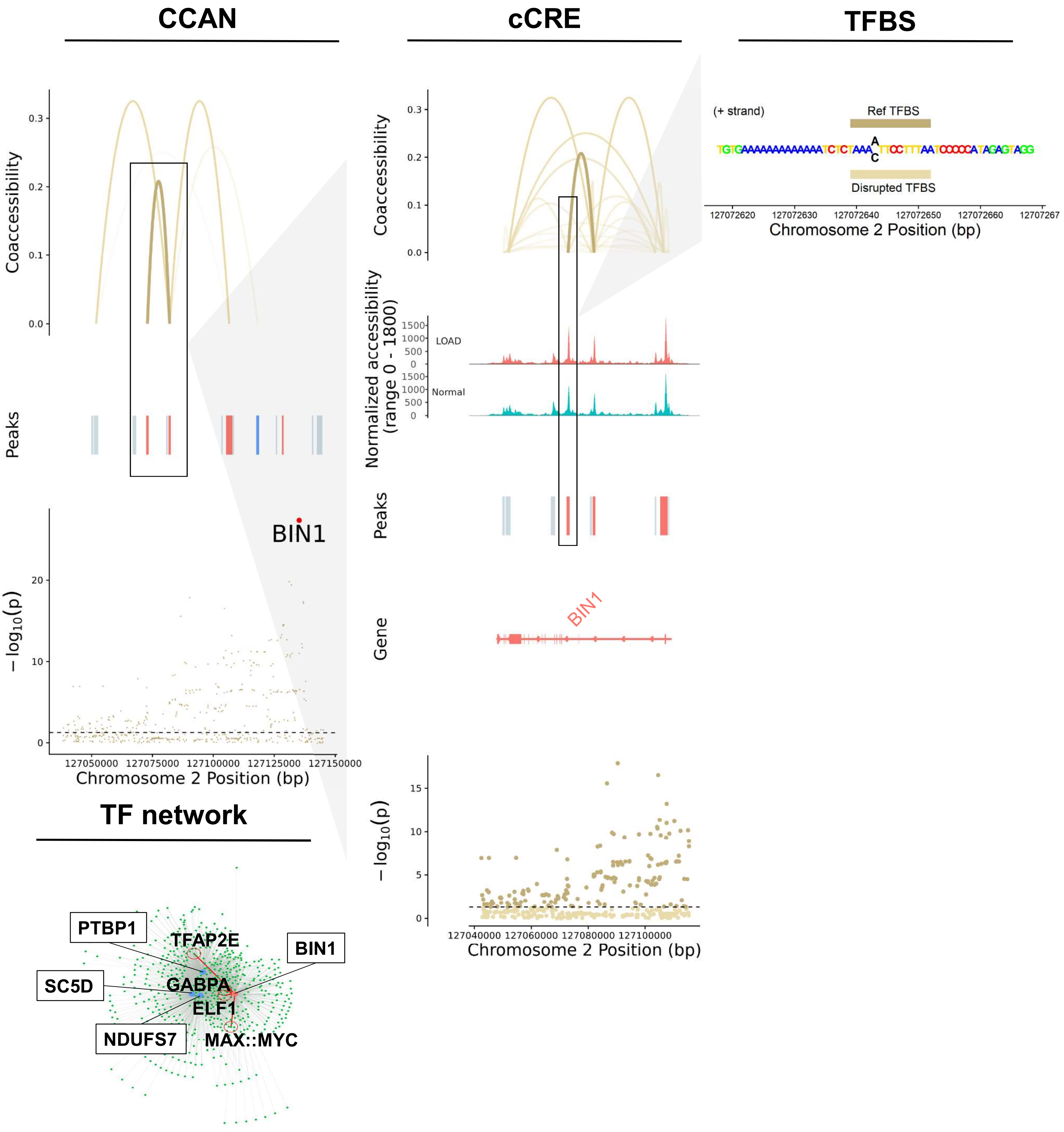

**Figure S7. Extended atSNP analysis**

**k**

## Oligo4, BIN1 // ELF1 (rs17014873)

Regulates membrane activity. Involved in cell cycle regulation and aptoptosis

| | |
|---|---|
| BIN1 (Intron 1) | ELF1 |
| *logFC = 0.15359, FDR = 0.036918* | *Affinity change = 4.6496, FDR = 0.0074374* |
| chr2-127072401-127073470 | rs17014873 |
| *logFC = 0.094153, FDR = 2.5135e-08* | *0.06606 (gnomAD), 0.1158 (BRAVO)* |

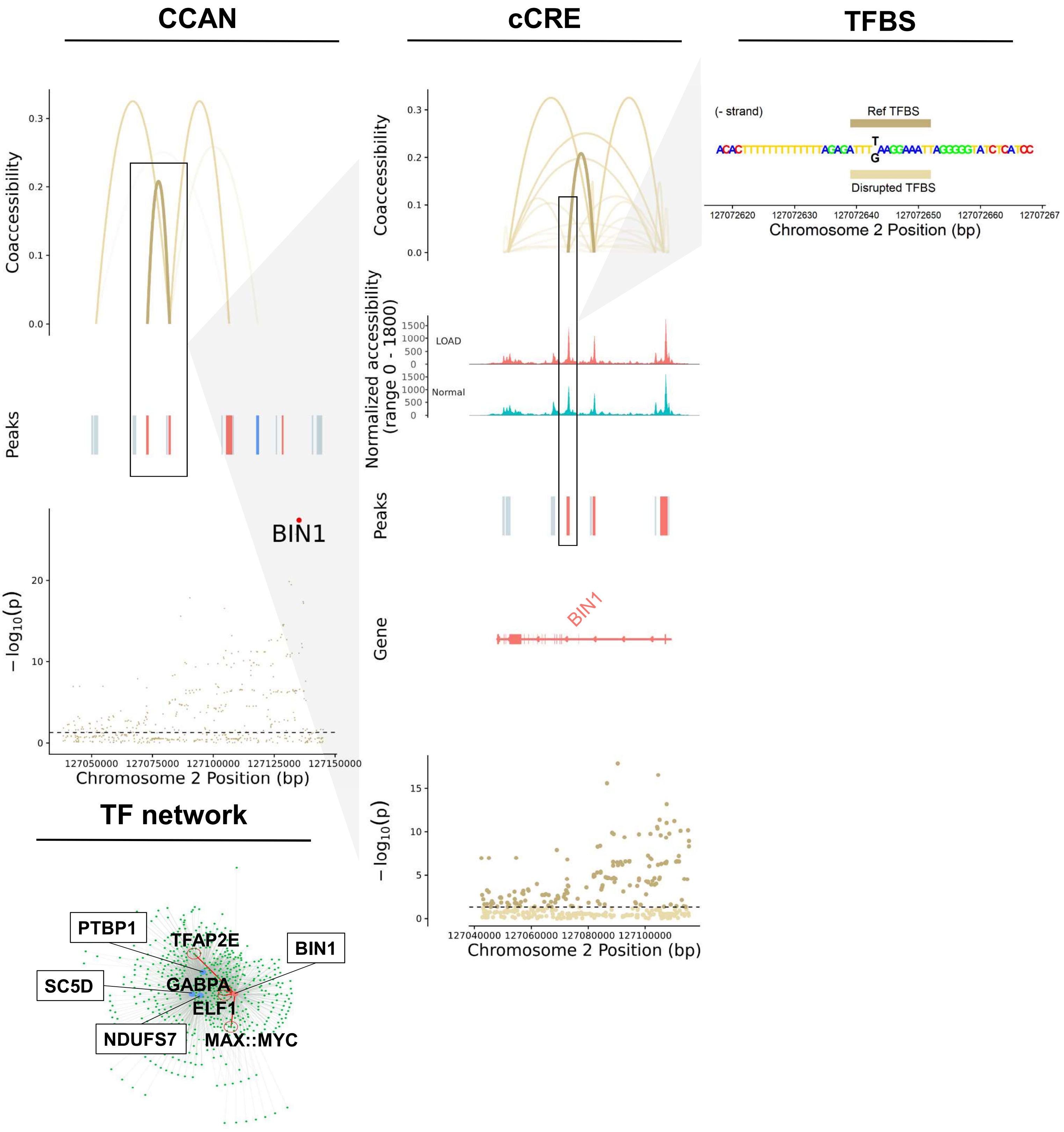

**Figure S7. Extended atSNP analysis**

## I

## Oligo4, BIN1 // MAX::MYC (rs72959057)

Regulates membrane activity. Involved in cell cycle regulation and aptoptosis

| | |
|---|---|
| BIN1 (Intron 1) | MAX::MYC |
| *logFC = 0.15359, FDR = 0.036918* | *Affinity change = 4.4427, FDR = 0.0023161* |
| chr2-127105247-127107901 | rs72959057 |
| *logFC = 0.082583, FDR = 3.3712e-05* | *0.05645 (gnomAD), 0.089017 (BRAVO)* |

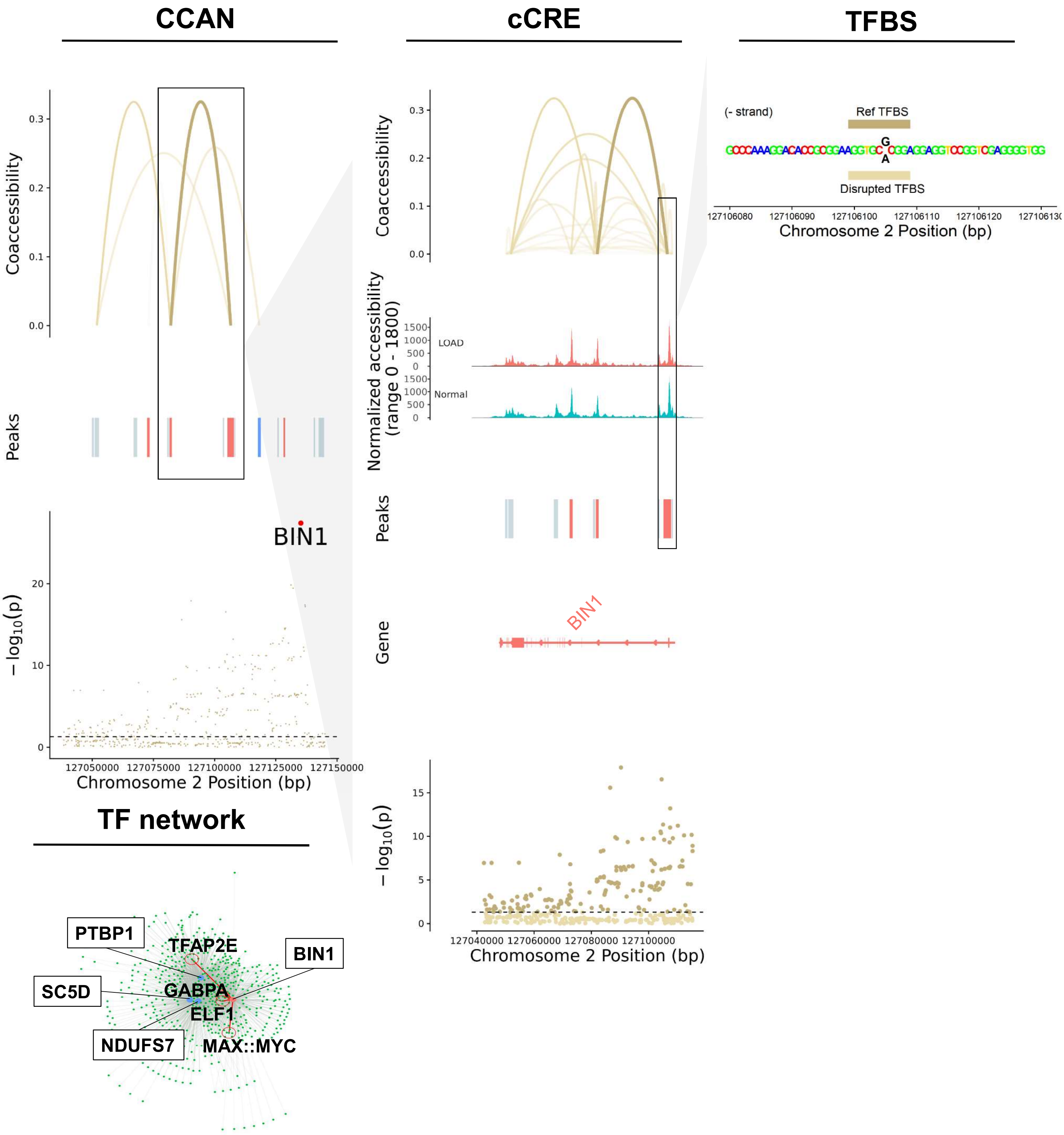

**Figure S7. Identification of SNPs predicted to influence TF binding affinity at GWAS loci in LOAD CCANs. a-l**, Diagrams of specific example SNP-TFBS overlaps. The cell subtype, regulated DEG, TF and SNP ID are shown in bold. The log fold change (*Log2FC*) and significance value (FDR) are shown for each DEG and corresponding cCRE. Additionally, functional information for each DEG is provided. The effect of the SNP on the TFBS affinity change and corresponding FDR determined using atSNP (see Methods) are noted. CCAN stacked plots show peak coaccessibility scores, directionality of changes in DAP accessibility in LOAD (red = increased accessibility, blue = reduced accessibility), and degree of LOAD association for GWAS loci. All features are arranged along the same horizontal access to indicate chromosomal position. cCRE stacked plots are detailed from boxed area of CCAN plots and additionally indicate overlapped gene coding regions, with upregulated DEGs shown in red and downregulated DEGs shown in blue, as well as normalized chromatin accessibility of the genomic region in LOAD and Normal samples. TFBS activity stacked plots are detailed from boxed areas of cCRE plots and indicate aligned chromosomal positions of TFBSs (Ref and disrupted TFBS - dark and light gold horizontal bars, respectively - were determined based on position weight matrix as described in Methods) and SNPs (black lettering). TF Network plots illustrate potential regulatory networks between DEG-overlapping peaks (blue) and TFBS-overlapping peaks (green), with those linkages predicted to be affected by LOAD SNPs shown in red.
